# Integrative Multiomics Analysis Reveals Tumor‐Associated Macrophage Heterogeneity and a Prognostic Signature in Gastric Cancer

**DOI:** 10.1155/humu/6130883

**Published:** 2026-05-27

**Authors:** Zhaoyan Li, Ming Xu, Yuqing Huang, Chen Huang, Yuan Wu, Jiafeng Lu, Guangtao Zhang, Lan Zheng

**Affiliations:** ^1^ Department of Traditional Chinese Medicine, Shanghai Jiao Tong University School of Medicine Affiliated Ruijin Hospital, Shanghai, China, shsmu.edu.cn; ^2^ Department of Gastrointestinal Surgery, RenJi Hospital, Shanghai Jiao Tong University School of Medicine, Shanghai, China, shsmu.edu.cn; ^3^ Department of Interventional Oncology, Seventh People′s Hospital of Shanghai University of Traditional Chinese Medicine, Shanghai, China

**Keywords:** gastric cancer, machine learning, single-cell RNA-sequencing, spatial transcriptomics, tumor-associated macrophages

## Abstract

Gastric cancer (GC) is characterized by a complex tumor microenvironment (TME) with substantial cellular heterogeneity. Tumor‐associated macrophages (TAMs) represent the most abundant immune cell population in the TME and exhibit remarkable functional plasticity. This study integrated single‐cell RNA‐sequencing (scRNA‐seq) data, bulk transcriptomics, and spatial transcriptomics to systematically characterize TAM heterogeneity and identify prognostic biomarkers in GC. ScRNA‐seq analysis revealed nine major cell types (T cells, plasma cells, epithelial cells, fibroblasts, macrophages, endothelial cells, B cells, smooth muscle cells, and mast cells) and distinct macrophage subpopulations with tumor‐specific expansion patterns. High‐dimensional weighted gene coexpression network analysis identified coexpression modules enriched in GC‐associated macrophages. Machine learning algorithms were employed to construct a prognostic signature, and the CoxBoost model demonstrated superior predictive performance across multiple cohorts. The seven‐gene signature, including UPP1, VCAN, ELL2, ABCA1, TUBA1A, MX2, and TSPO, showed robust prognostic value in survival prediction. Spatial transcriptomic analysis further revealed distinct metabolic profiles and extensive cellular interaction networks mediated by UPP1‐expressing TAMs. These findings provide a comprehensive atlas of TAM heterogeneity and establish novel prognostic biomarkers with potential therapeutic implications in GC.

## 1. Introduction

Gastric cancer (GC) represents one of the most prevalent malignancies worldwide, ranking as the fifth most common cancer and the fourth leading cause of cancer‐related mortality globally [1, 2]. Despite advances in surgical techniques, chemotherapy, and targeted therapies, the prognosis for advanced GC patients remains poor, with 5‐year survival rates below 30% [3, 4]. This clinical challenge underscores the urgent need to identify novel prognostic biomarkers and therapeutic targets through comprehensive characterization of the tumor microenvironment (TME).

The TME plays a pivotal role in tumor progression, metastasis, and therapeutic resistance [5, 6]. Among the diverse cellular constituents of the TME, tumor‐associated macrophages (TAMs) constitute the most abundant immune cell population and exhibit remarkable functional plasticity [7]. TAMs are generally classified into proinflammatory M1‐like and anti‐inflammatory M2‐like phenotypes; however, emerging single‐cell transcriptomic studies have revealed substantial heterogeneity beyond this binary classification. In GC, TAMs have been implicated in promoting tumor angiogenesis, immune evasion, and metastatic dissemination [8–10]. Nevertheless, the precise molecular programs governing TAM heterogeneity and their clinical relevance in GC remain incompletely understood.

Recent advances in single‐cell technologies and machine learning algorithms have enabled systematic identification of gene modules and construction of robust prognostic models from complex transcriptomic data. These computational approaches offer unprecedented opportunities to dissect cellular heterogeneity and discover clinically actionable signatures. Integrative analysis of single‐cell RNA sequencing (scRNA‐seq) with spatial transcriptomics further allows for the mapping of cellular interactions within intact tissue architecture, providing critical insights into the spatial organization of the TME [11]. However, the application of these multiomics approaches to systematically characterize TAM subpopulations and their metabolic features in GC remains limited.

Metabolic reprogramming represents a hallmark of cancer and is increasingly recognized as a critical regulator of immune cell function within the TME [5]. TAMs undergo substantial metabolic adaptations that shape their polarization states and functional properties, including altered glycolysis, lipid metabolism, and amino acid utilization. Understanding the metabolic heterogeneity of TAM subpopulations may reveal novel therapeutic vulnerabilities and prognostic indicators [12–14]. Spatial transcriptomic technologies now enable the mapping of metabolic pathway activities within distinct tissue regions, offering insights into the metabolic crosstalk between TAMs and neighboring malignant or stromal cells.

In this study, we integrated scRNA‐seq, bulk transcriptomic, and spatial transcriptomic data to characterize TAM heterogeneity in GC. Through bioinformatics analyses and machine learning‐based prognostic modeling, we identified a macrophage‐related gene signature associated with adverse clinical outcomes. Furthermore, spatial analysis revealed distinct metabolic profiles and cellular interaction networks mediated by specific TAM subpopulations. Our findings provide a comprehensive atlas of TAM heterogeneity and establish novel prognostic biomarkers with potential therapeutic implications in GC.

## 2. Materials and Methods

### 2.1. Data Acquisition and Preprocessing

Publicly available scRNA‐seq data of GC were obtained from the Gene Expression Omnibus (GEO) database under accession number GSE183904 [15], which included five paired GC and adjacent normal tissue samples. For spatial transcriptomic analysis, data were retrieved from GSE251950 [16]. Bulk RNA‐seq data and corresponding clinical information for prognostic validation were downloaded from The Cancer Genome Atlas Stomach Adenocarcinoma (TCGA‐STAD) cohort [17] and four independent GEO datasets (GSE15459, GSE26253, GSE26901, and GSE28541) [18, 19].

For scRNA‐seq data preprocessing, raw count matrices were imported into R (Version 4.2.0) and analyzed using the Seurat package (Version 4.0). Quality control was performed to exclude cells with fewer than 200 detected genes, more than 10,000 detected genes, or mitochondrial gene content exceeding 20%. Doublet removal was conducted using the DoubletFinder algorithm. Data normalization was performed using the LogNormalize method with a scale factor of 10,000, followed by identification of highly variable features using the FindVariableFeatures function with selection method “vst” and 2000 features. Data integration across samples was performed using the Harmony algorithm to correct batch effects. Principal component analysis (PCA) was conducted on the scaled data, and the first 30 principal components were used for Uniform Manifold Approximation and Projection (UMAP) dimensionality reduction with default parameters.

### 2.2. Cell Type Annotation and Subpopulation Analysis

Cell clusters were initially annotated using canonical marker genes obtained from published literature and the CellMarker database. The major cell populations identified included T cells (CD3D and CD3E), B cells (CD79A and MS4A1), plasma cells (IGHG1 and IGLC2), macrophages (CD68, CD14, and CD163), fibroblasts (COL1A1 and COL1A2), endothelial cells (PECAM1 and VWF), epithelial/malignant cells (EPCAM and KRT18), mast cells (TPSAB1 and CPA3), and smooth muscle cells (ACTA2 and MYH11).

For macrophage subpopulation analysis, CD68‐positive cells were extracted from the integrated dataset and subjected to reclustering using the same pipeline described above. Differential abundance analysis between tumor and normal tissues was performed using the Milo algorithm with a false discovery rate (FDR) threshold of 0.05. Macrophage clusters significantly expanded in tumor tissues (Clusters 1, 2, 6, and 10) were designated as GC‐associated macrophages (GC_Macro), whereas remaining clusters were classified as Other_Macro.

### 2.3. Cell–Cell Communication Analysis

Intercellular communication analysis was performed using the CellChat package (Version 1.6.0) with the human ligand‐receptor interaction database [20]. Communication probabilities were calculated using the “triMean” method for secretion signaling analysis. The number of interactions and interaction weights were computed and visualized as circle plots. Significant ligand‐receptor pairs were identified with a minimum communication probability threshold of 0.05 and *p* value < 0.05. Outgoing and incoming signaling patterns were analyzed using the netAnalysis_computeCentrality and netAnalysis_signalingRole functions, respectively. For spatial transcriptomic data, cell–cell interactions were inferred using the SpaTalk algorithm with default parameters across three spatial scales: intraspot (same spatial coordinate), juxta‐5 (neighboring five spots), and para‐15 (distant 15 spots).

### 2.4. High‐Dimensional Weighted Gene Coexpression Network Analysis (hdWGCNA)

hdWGCNA was performed on macrophage subpopulations using the hdWGCNA package [21]. Soft‐thresholding power selection was conducted using the pickSoftThreshold function with power values ranging from 1 to 30. The optimal soft‐thresholding power was determined based on the scale‐free topology fit index (*R* ^2^ > 0.8) and mean connectivity. Signed coexpression networks were constructed using the blockwiseModules function with the following parameters: maxBlockSize = 10,000, deepSplit = 4, minModuleSize = 50, mergeCutHeight = 0.15. Module eigengenes (MEs) were calculated as the first principal component of each module. Module membership (kME) was defined as the correlation between gene expression and ME. Hub genes were identified as the Top 10 genes with highest kME values in each module. Module preservation analysis was performed to assess module stability across datasets.

### 2.5. Machine Learning‐Based Prognostic Model Construction

One hundred fifty candidate genes were selected from the red, blue, and green modules (Top 50 genes per module based on kME values) for prognostic modeling. Uunivariate Cox regression analysis was performed to identify genes significantly associated with overall survival (OS) using the survival package (*p* < 0.05). Ten machine learning algorithms were compared for model construction: CoxBoost, stepwise Cox regression (backward and forward), Ridge regression, Elastic Net regression (*α* = 0.1–0.9, increments of 0.1), and Lasso regression. For each algorithm, fivefold cross‐validation was performed to optimize hyperparameters. Model performance was evaluated using time‐dependent area under the receiver operating characteristic curve (AUC) at 1, 3, and 5 years with the timeROC package. The optimal model was selected based on the highest average AUC across the TCGA‐STAD and four GEO validation cohorts.

Risk scores were calculated for each patient using the formula: Risk Score = *Σ* (Coefficient_i_ × Expression_i_), where Coefficient_i_ represents the gene coefficient derived from the CoxBoost algorithm and Expression_i_ represents the normalized expression level of gene *i*. Patients were stratified into high‐risk and low‐risk groups using the median risk score as the cutoff. Kaplan–Meier survival analysis was performed using the survminer package, and statistical significance was assessed using the log‐rank test. Multivariate Cox regression analysis was conducted to evaluate the independent prognostic value of the risk score adjusting for clinical covariates (age, sex, stage, and Lauren classification). Meta‐analysis of hazard ratios (HRs) across cohorts was performed using the meta package with a random‐effects model.

### 2.6. Spatial Transcriptomic Data Analysis

Spatial transcriptomic data preprocessing was performed using the Seurat and SpatialExperiment packages. Quality control filtering excluded spots with fewer than 500 detected genes or mitochondrial content exceeding 30%. Data normalization was conducted using SCTransform. Dimensionality reduction and clustering were performed using PCA and UMAP as described for scRNA‐seq data. Spatial feature plots were generated to visualize gene expression and cluster distributions across tissue sections. Cell type deconvolution of spatial transcriptomic data was performed using the SPOTlight algorithm with scRNA‐seq data as the reference. Metabolic pathway activity was inferred using the scMetabolism package [22]. Pathway activity scores were calculated using the Vision algorithm with default parameters. Spatial autocorrelation analysis was performed using Moran′s I statistic to assess the spatial clustering of metabolic activities. This analytical framework is consistent with recent spatial multiomics studies that characterized tumor‐stroma boundary cell features to predict breast cancer progression and therapy response [23].

### 2.7. Statistical Analysis

All statistical analyses were performed in R Version 4.2.0. Continuous variables were compared using Student′s *t*‐test or Wilcoxon rank‐sum test as appropriate. Categorical variables were compared using chi‐square test or Fisher′s exact test. Correlation analysis was performed using Pearson or Spearman correlation coefficients. Multiple testing correction was performed using the Benjamini–Hochberg method. Statistical significance was defined as *p* < 0.05.

## 3. Results

### 3.1. Single‐Cell Atlas of GC Reveals Distinct Macrophage Subpopulations

To investigate the cellular heterogeneity in GC, we analyzed the GSE183904 scRNA‐seq dataset. UMAP visualization identified nine major cell types, including T cells, plasma cells, epithelial cells, fibroblasts, macrophages, endothelial cells, B cells, smooth muscle cells, and mast cells (Figure 1A). Analysis of cellular composition across five paired samples (GC tissues versus normal tissues [NC]) revealed distinct distribution patterns between tumor and normal microenvironments (Figure 1B). The top five marker genes for each cell type were visualized in a dot plot, demonstrating the specific expression signatures of each cluster (Figure 1C).

Figure 1Single‐cell transcriptomic atlas reveals cellular heterogeneity and macrophage subpopulations in gastric cancer. (A) UMAP visualization identifies nine major cell types from five‐paired gastric cancer and normal tissues. (B) UMAP plots show distinct cellular composition between normal and tumor tissues. (C) Dot plot displays the top five marker genes defining each cell type. (D) Reclustering of macrophages reveals distinct subpopulations in normal and tumor tissues. (E) Differential abundance analysis identifies Clusters 1, 2, 6, and 10 as significantly expanded in tumor tissues (the absolute values of cell population in tumor and normal tissues are compared). (F) Circle plots depict the number and strength of intercellular interactions among all cell types. (G) Bubble plot shows significant ligand‐receptor pairs between different cell populations. (H) Heatmap reveals cell type‐specific outgoing and incoming signaling patterns. (I) Scatter plot demonstrates that macrophages exhibit distinct communication strengths compared with other cell types.

**Figure:**
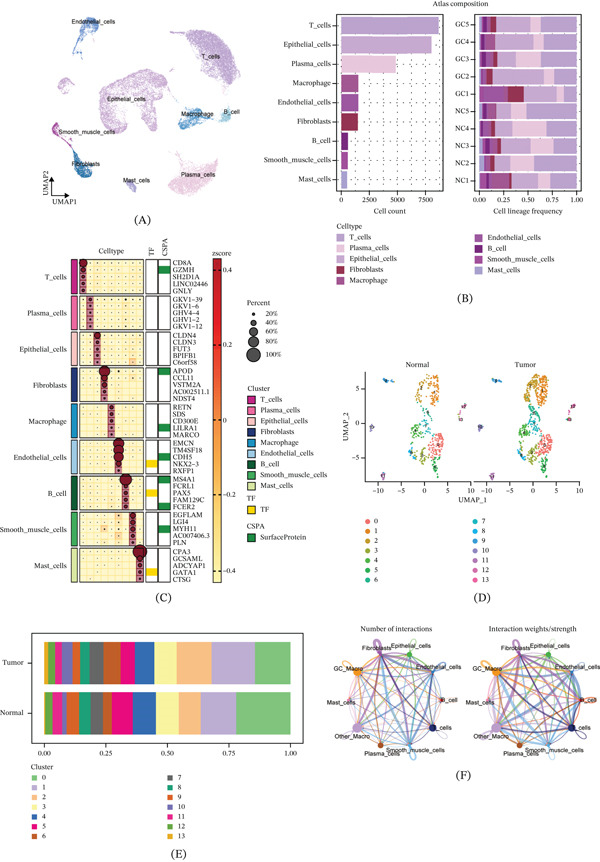

**Figure:**
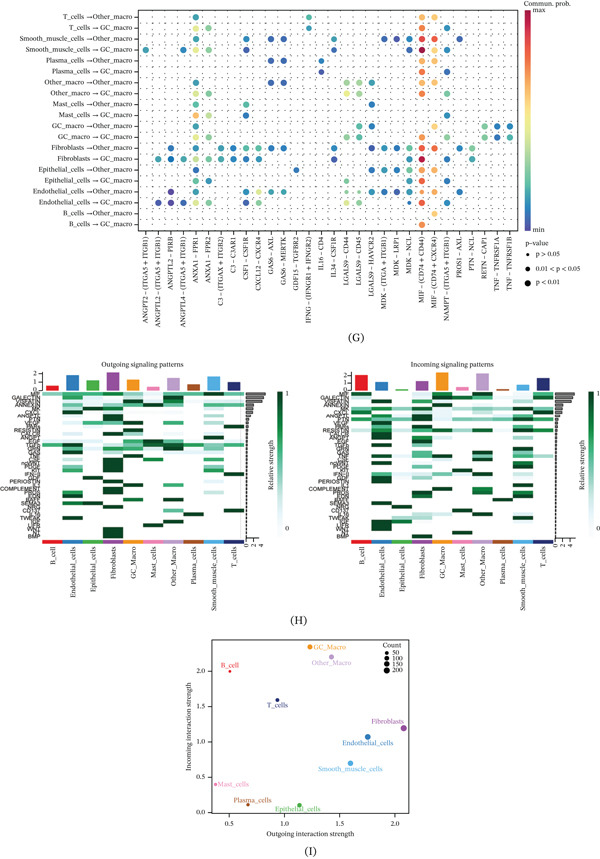

To further characterize macrophage heterogeneity, we extracted macrophages from the overall dataset and performed secondary clustering. UMAP visualization revealed distinct distribution patterns between normal and tumor groups (Figure 1D). Comparative analysis of cluster proportions between the two groups identified Clusters 1, 2, 6, and 10 as significantly expanded in tumor tissues, which we designated as GC‐associated macrophages (GC_Macro), whereas the remaining clusters were classified as Other_Macro (Figure 1E).

Cell–cell communication analysis revealed extensive interactions among different cell types. The network analysis showed both the number of interactions and interaction weights/strength among various cell populations (Figure 1F). Bubble plots of significant ligand‐receptor pairs demonstrated distinct communication probabilities between different cell types, with dot size indicating statistical significance (Figure 1G). Analysis of outgoing and incoming signaling patterns revealed cell type‐specific communication profiles (Figure 1H). Furthermore, scatter plot analysis of overall communication strength showed that macrophages exhibited distinct patterns of outgoing and incoming signal intensities compared with other cell types (Figure 1I).

### 3.2. Identification of Macrophage Signature Genes Through hdWGCNA Analysis

To identify gene modules associated with GC‐specific macrophages, we performed hdWGCNA. Soft‐threshold power selection analysis indicated an optimal power value for constructing scale‐free networks, as evidenced by the scale‐free topology fit index, mean connectivity, and signed network metrics (Figure 2A). Hierarchical clustering dendrogram based on the topological overlap matrix (TOM) revealed distinct coexpression modules, represented by different colors (Figure 2B).

Figure 2High‐dimensional weighted gene coexpression network analysis identifies macrophage module genes associated with gastric cancer. (A) Soft‐thresholding power analysis determines the optimal power value for scale‐free network construction. (B) Hierarchical clustering dendrogram reveals nine distinct coexpression modules based on topological overlap. (C) Module eigengene analysis lists the Top 10 hub genes for the red, blue, and green modules. (D) Correlation heatmap shows the relationships between module eigengenes. (E) UMAP visualization demonstrates specific enrichment patterns of the red, blue, and green modules across macrophage subpopulations. (F) Bubble plot confirms that the red, blue, and green modules are specifically enriched in GC‐expanded macrophage clusters.

**Figure:**
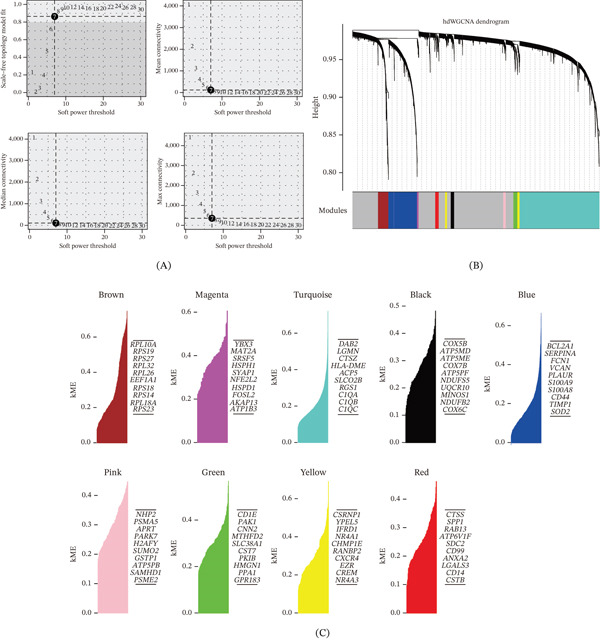

**Figure:**
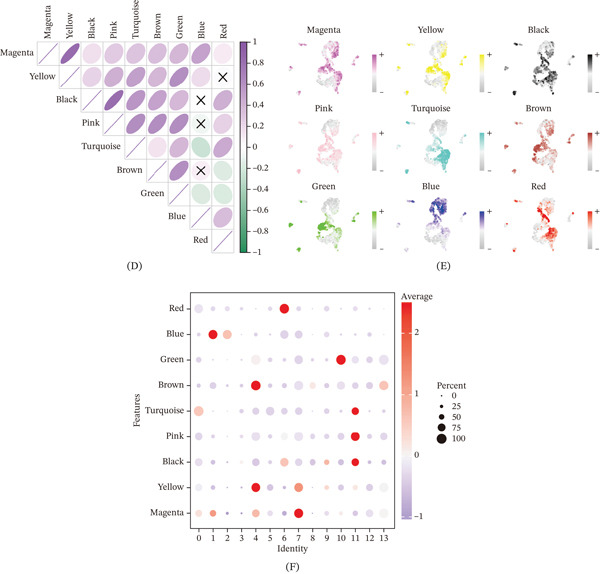

MEs analysis identified nine coexpression modules (brown, magenta, turquoise, black, blue, pink, green, yellow and red). For each module, we visualized the distribution of module membership (kME) values and listed the top 10 hub genes ranked by kME (Figure 2C). Correlation analysis between modules showed varying degrees of relationships, with some modules exhibiting strong positive correlations whereas others showed no significant association (Figure 2D). Spatial visualization of MEs on the UMAP plot revealed distinct expression patterns among macrophage subpopulations (Figure 2E). Bubble plot analysis of ME expression across different cell clusters indicated that the red, blue, and green modules were specifically enriched in the GC‐expanded macrophage clusters (Clusters 1, 2, 6, and 10). Based on these findings, we selected the top 50 genes from each of these three modules for subsequent prognostic analysis (Figure 2F).

### 3.3. Machine Learning‐Based Construction of a Macrophage‐Related Prognostic Signature

From the 150 candidate genes derived from hdWGCNA analysis, we performed univariate Cox regression analysis and identified 16 genes significantly associated with OS. Forest plot visualization revealed their HRs and 95% confidence intervals (CIs), with UPP1, FCGR2A, and SDC2 showing the highest risk associations (HR > 1.3) (Figure 3A).

Figure 3Machine learning‐based construction and validation of a macrophage‐related prognostic gene signature for gastric cancer. (A) Forest plot displays univariate Cox regression results for 16 genes significantly associated with overall survival. (B) Bar plot compares the predictive performance of 10 machine learning algorithms across multiple cohorts. (C) Coefficient values show that UPP1 contributes most significantly to the CoxBoost prognostic model. (D) Meta‐analysis forest plot demonstrates a combined hazard ratio of 1.9107 across seven independent cohorts. (E) Kaplan–Meier survival curves consistently show that high‐risk patients have significantly worse outcomes across all cohorts. (F) UPP1 is significantly elevated in GC compared with normal tissues (*p* = 0.00053). (G) The correlation heatmap shows the correlations between UPP1 expression and each step of cancer immunity cycle in gastric cancer. (H) Immune feature scores in GC samples with different UPP1 expression levels. (I) Considerable variation in the expression, CNV, and epigenetic alterations of most immune‐related genes with different UPP1 expression levels in the TCGA‐STAD cohort.

**Figure:**
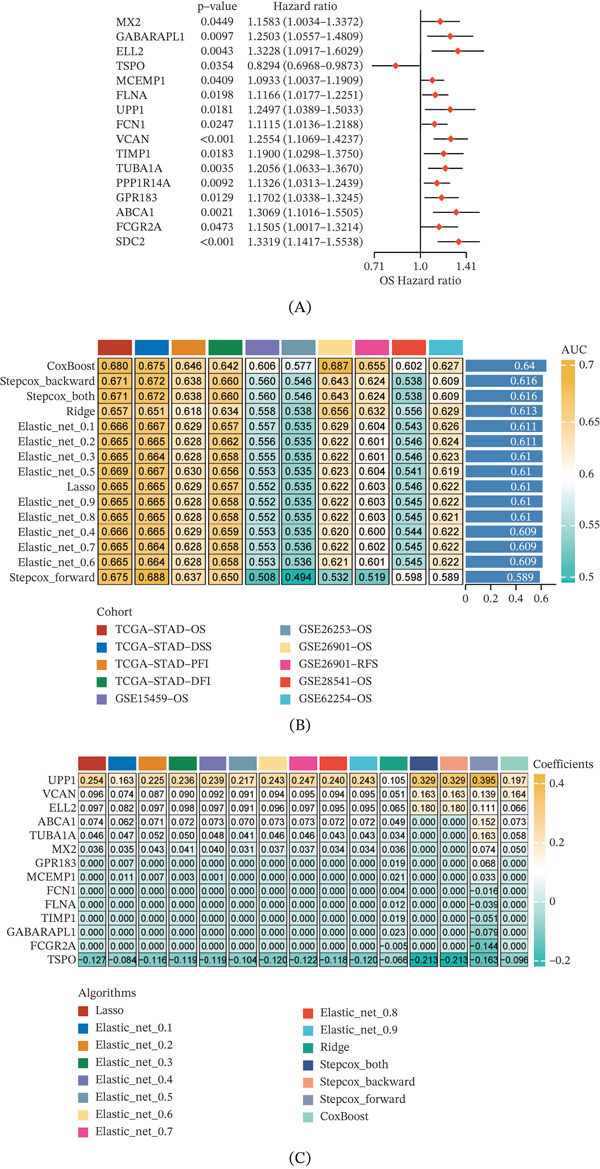

**Figure:**
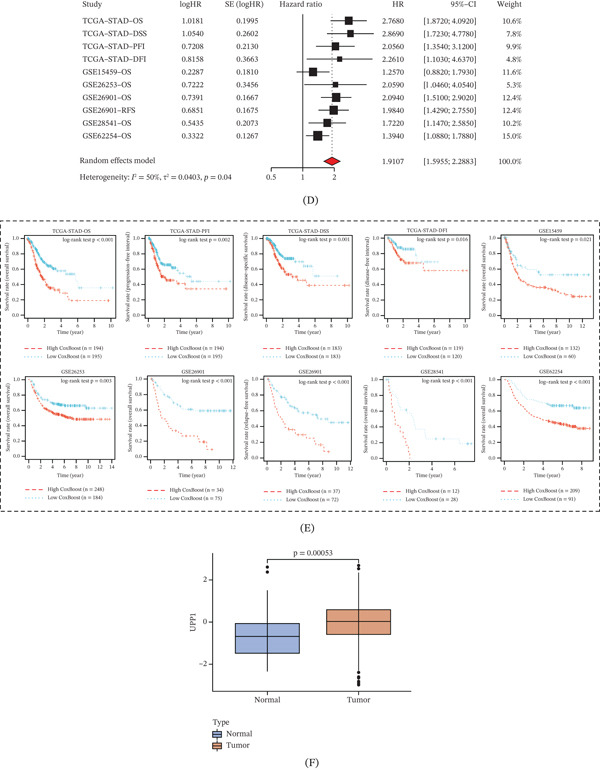

**Figure:**
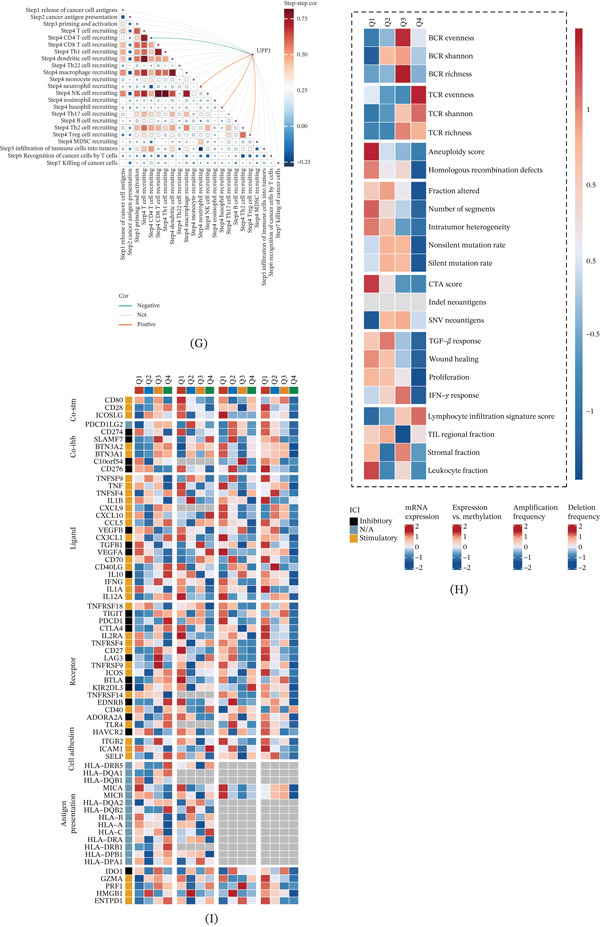

We compared 10 different machine learning algorithms for prognostic model construction, including CoxBoost, StepCox (backward and forward), Ridge regression, Elastic Net (various alpha values), and Lasso regression. Evaluation of 1‐, 3‐, and 5‐year area under the curve (AUC) values across multiple cohorts (TCGA‐STAD and four GEO datasets) demonstrated that CoxBoost achieved the highest average AUC (0.680 at 1‐year, 0.687 at 3‐year, and 0.655 at 5‐year), indicating superior predictive performance (Figure 3B). Analysis of gene coefficients across different algorithms revealed that UPP1 consistently showed the highest coefficient in the CoxBoost model, followed by VCAN, ELL2, ABCA1, TUBA1A, MX2, and TSPO (Figure 3C).

Meta‐analysis of the prognostic model across seven cohorts confirmed the robustness of our signature, with a combined HR of 1.9107 (95% CI: 1.5955–2.2883) and moderate heterogeneity (*I* ^2^ = 50*%*) (Figure 3D). Kaplan–Meier survival analysis consistently demonstrated that patients in the high‐risk group had significantly worse OS, disease‐free survival, and progression‐free interval compared with the low‐risk group across all cohorts (*p* < 0.05 for all comparisons) (Figure 3E).

Subsequently, we analyzed UPP1 expression in GC and normal tissues, and found that UPP1 was significantly elevated in GC tissues (*p* = 0.00053; Figure 3F). The correlation heatmap illustrates the correlation between UPP1 gene expression and the cancer‐immunity cycle steps in GC tissues, where red indicates positive correlation and blue indicates negative correlation; the arcs on the right show that UPP1 is positively correlated (green lines) with the recruitment of multiple effector immune cells including CD8+ T cells, Th1 cells, and NK cells, whereas negatively correlated (orange lines) with immunosuppressive cells such as Tregs and MDSCs (Figure 3G). This seemingly complex pattern—effector cell enrichment alongside poor prognosis—suggests a context‐dependent immune landscape rather than a simple pro‐ or anti‐inflammatory dichotomy (see Discussion).

Furthermore, we analyzed the immune feature scores in GC samples with different UPP1 expression levels, and revealed substantial heterogeneity in immune infiltration patterns between high and low UPP1 expression groups (Figure 3H). Considerable variation in the expression, copy number variation (CNV), and epigenetic alterations of most immune‐related genes across GC samples with different UPP1 expression levels was observed in the TCGA‐STAD cohort, indicating that UPP1 expression status is associated with widespread heterogeneity in the tumor immune microenvironment (Figure 3I).

### 3.4. Characterization of UPP1^+^ TAMs and Their Cellular Interactions

Focusing on the TME, we reclustered the tumor samples (excluding normal tissues) and identified nine cell types: T cells, plasma cells, malignant cells, TAMs, cancer‐associated fibroblasts (CAF), tumor endothelial cells (TEC), mast cells, B cells, and smooth muscle cells (Figure 4A). Feature plot analysis revealed that UPP1 was predominantly expressed in the TAM population (Figure 4B).

Figure 4Characterization of UPP1^+^ tumor‐associated macrophages and their intercellular communication networks. (A) UMAP visualization identifies nine major cell types in tumor samples. (B) Feature plot shows that UPP1 is predominantly expressed in the TAM population. (C) Reclustering of TAMs reveals distinct subpopulations with heterogeneous gene expression. (D) Feature plot enables classification of UPP1‐high and UPP1‐low TAM subgroups. (E) Circle plots reveal enhanced interactions of UPP1^+^ TAM with malignant cells, CAF, and TEC. (F) Heatmap shows ligand‐receptor interactions between UPP1^+^ TAM and malignant cells. (G) Bubble plot depicts angiogenesis‐related interactions between UPP1^+^ TAM and TEC. (H) Bubble plot demonstrates immunomodulatory interactions between UPP1^+^ TAM and T cells.

**Figure:**
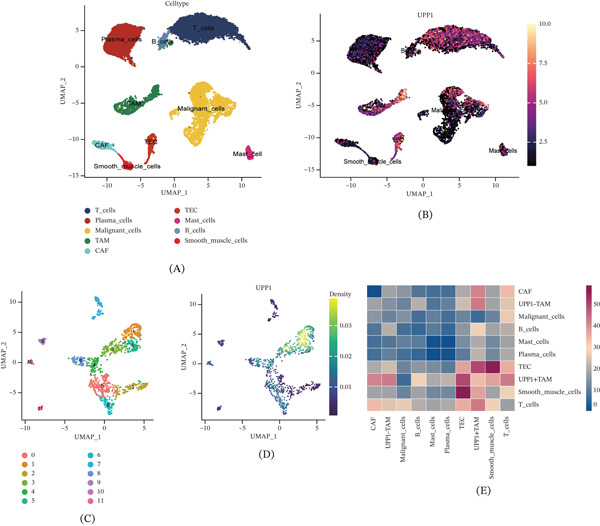

**Figure:**
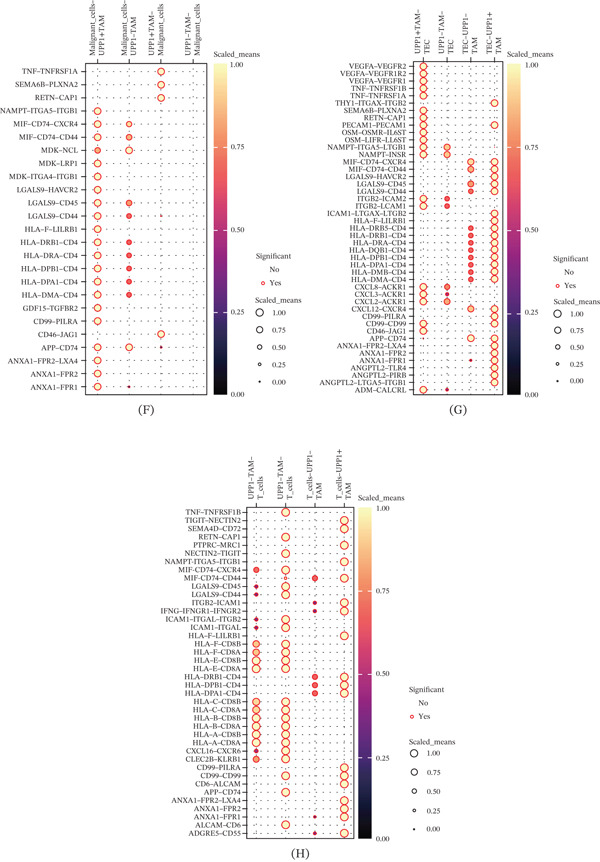

Subclustering of TAMs identified distinct subpopulations with heterogeneous UPP1 expression patterns (Figure 4C,D). Density plot analysis enabled the classification of UPP1‐high and UPP1‐low TAM subgroups. Cell–cell communication analysis comparing UPP1^+^ TAM versus UPP1^−^ TAM revealed distinct communication patterns with other cell types, with UPP1^+^ TAM showing enhanced interactions with malignant cells, CAF, and TEC (Figure 4E). Notably, CAFs have been reported to regulate cancer stem cell stemness through the Wnt/*β*‐catenin and SDF‐1/CXCR4 pathways [24], suggesting that the CAF‐TAM crosstalk may contribute to GC progression via analogous mechanisms.

Detailed analysis of ligand‐receptor interactions showed that UPP1^+^ TAM exhibited specific communication patterns with malignant cells through various signaling pathways (Figure 4F). Notably, UPP1^+^ TAM demonstrated enhanced interactions with TEC through angiogenesis‐related ligand‐receptor pairs (Figure 4G). Furthermore, UPP1^+^ TAM showed distinct immunomodulatory interactions with T cells, suggesting potential roles in immune regulation (Figure 4H).

### 3.5. Spatial Transcriptomic Analysis Reveals Metabolic Heterogeneity in GC

To investigate the spatial organization of GC, we analyzed spatial transcriptomic data from GSE251950. UMAP visualization of spatial spots identified 14 distinct clusters with unique spatial distribution patterns (Figure 5A,B). Analysis of TAM signature gene expression across spatial clusters revealed that UPP1, TSPO, and FCGR2A were enriched in specific spatial regions (Figure 5C).

Figure 5Spatial transcriptomic analysis reveals metabolic heterogeneity and tissue organization in gastric cancer. (A) UMAP visualization identifies 14 distinct spatial clusters across the tissue section. (B) Spatial feature plots show region‐specific localization patterns of selected clusters. (C) Spatial expression patterns reveal enrichment of TAM signature genes in specific tumor regions. (D) Heatmap demonstrates distinct metabolic pathway enrichment across spatial clusters. (E) Spatial visualization shows heterogeneous distribution of galactose metabolism activity. (F) Spatial visualization reveals region‐specific enrichment of glycerophospholipid metabolism.

**Figure:**
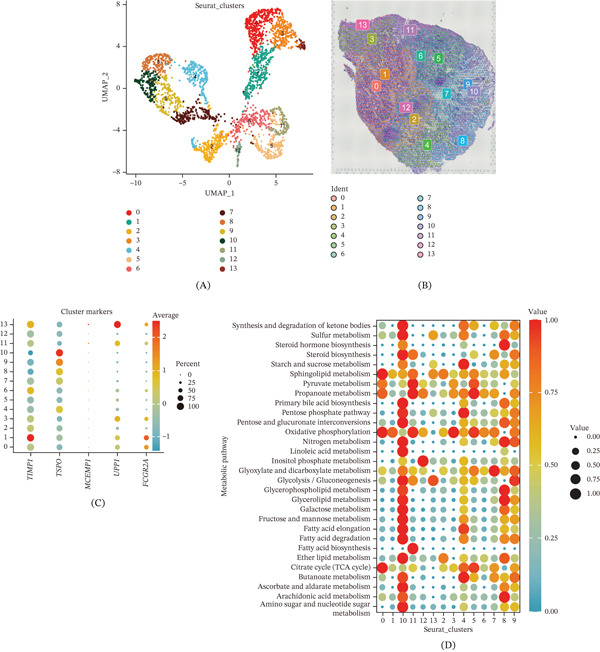

**Figure:**
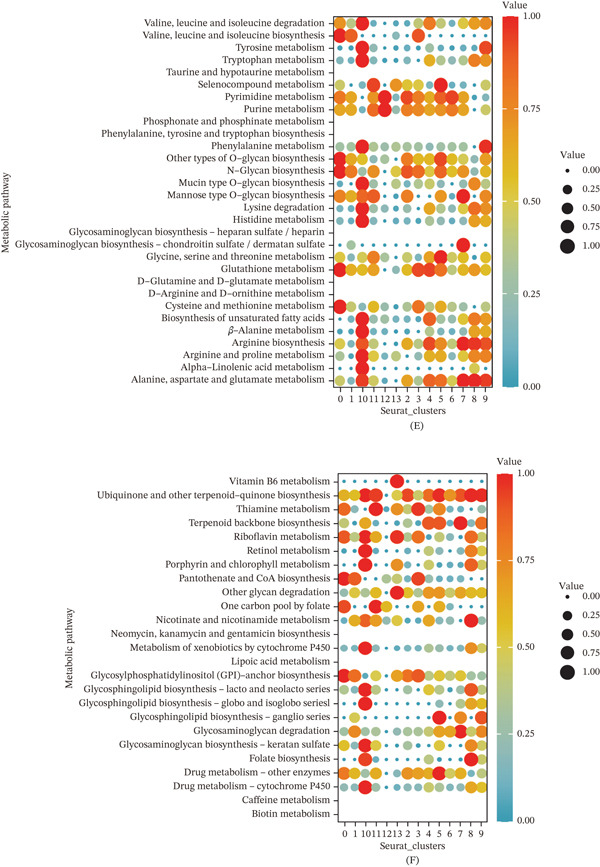

Metabolic pathway enrichment analysis across spatial clusters revealed distinct metabolic profiles. Clusters exhibited differential enrichment in various metabolic pathways, including glycolysis, gluconeogenesis, amino acid metabolism, and lipid metabolism (Figure 5D–F). Notably, certain clusters showed high activity in galactose metabolism and glycerophospholipid metabolism, suggesting metabolic heterogeneity within the TME.

### 3.6. Spatial Deconvolution Reveals UPP1^+^ TAM‐Mediated Cellular Networks

Spatial feature plot visualization demonstrated distinct UPP1 expression patterns across the tissue section, with concentrated expression in specific tumor regions (Figure 6A). Analysis of cell type enrichment revealed the spatial distribution of various cell populations, including malignant cells, TAM subsets, and stromal components (Figure 6B).

Figure 6Spatial deconvolution identifies UPP1^+^ TAM‐mediated cellular interaction networks in the gastric cancer microenvironment. (A) Spatial feature plot shows concentrated UPP1 expression in specific tumor regions. (B) Spatial maps display the distribution of malignant cells, TAM subsets, and stromal components. (C) Ranking plot identifies malignant cells as having the strongest spatial association with UPP1^+^ TAM. (D) Bar plot shows cell type contributions to spatial interactions across three spatial scales. (E–G) Network visualizations reveal distinct interaction patterns at intraspot, juxta‐5, and para‐15 spatial scales. (H) Comprehensive spatial mapping confirms colocalization of UPP1^+^ TAM with malignant and immune cells. (I) Dot plot identifies SPON2‐ITGB1 as the top‐ranked ligand‐receptor pair. (J) Spatial visualization reveals concentrated SPON2‐ITGB1 signaling hotspots. (K) Dot plot shows the strength of individual ligand–receptor (L–R) pairs across cell–cell interactions. (L) Dot plot illustrates the overall sender/receiver signaling strength between cell subpopulations. (M) Integrated network analysis illustrates the central role of UPP1^+^ TAM in orchestrating cellular communications.

**Figure:**
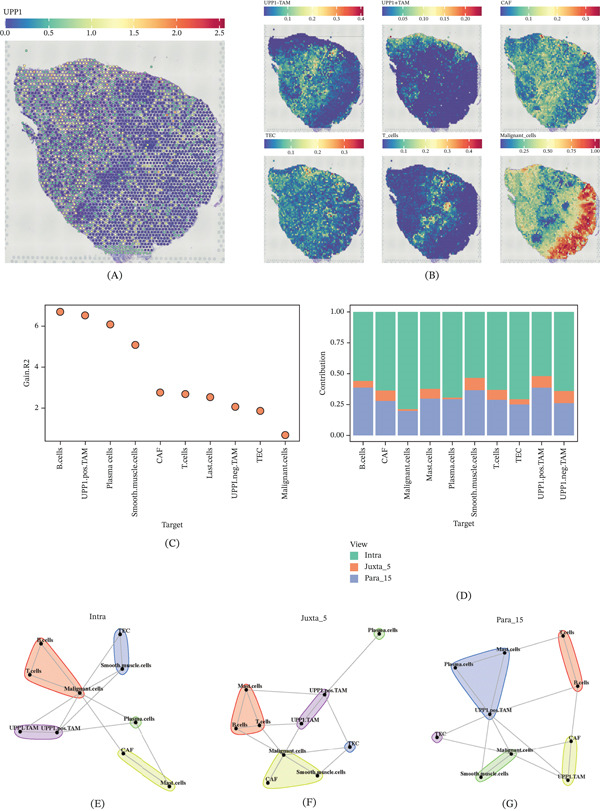

**Figure:**
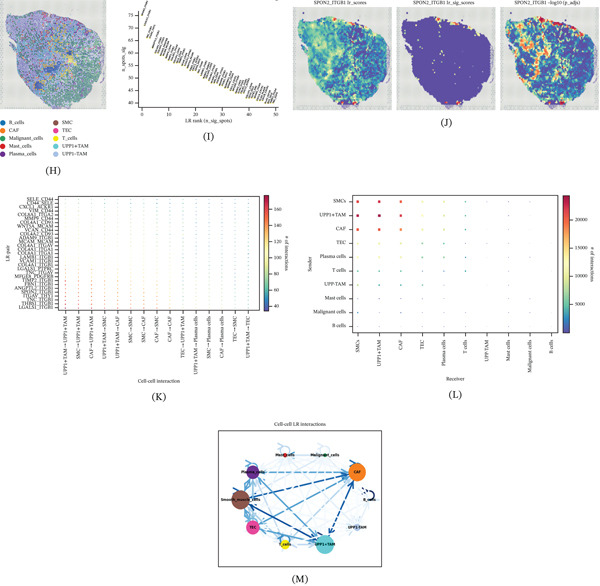

Spatial interaction analysis ranked different target cell types by their spatial association significance. UPP1^+^ TAM showed the strongest spatial interactions with malignant cells and other immune components (Figure 6C). Multiscale spatial interaction analysis revealed that different cell types contributed variably across spatial scales: intraspot (within the same spot), juxta‐5 (neighboring five spots), and para‐15 (distant 15 spots) (Figure 6D). Network visualization at each spatial scale demonstrated distinct interaction patterns, with UPP1^+^ TAM forming tight interaction modules at the intraspot level and extending communications at broader spatial ranges (Figure 6E–G).

Comprehensive spatial cell type mapping confirmed the colocalization of UPP1^+^ TAM with malignant and immune cells (Figure 6H). Ligand‐receptor analysis identified the Top 50 significant pairs, with SPON2‐ITGB1 ranking highest (Figure 6I). Spatial visualization of SPON2‐ITGB1 interaction scores revealed concentrated signaling hotspots (Figure 6J). Integrin‐mediated signaling is a well‐established driver of cancer cell migration and invasion; for instance, osthole has been shown to inhibit the migration and invasion of highly metastatic breast cancer cells by suppressing ITG*α*3/ITG*β*5 signaling [25], supporting the functional relevance of the SPON2‐ITGB1 axis in the GC TME. Cell–cell interaction heatmaps demonstrated extensive ligand‐receptor communications across the tissue (Figure 6K,L). Finally, network analysis integrating UPP1^+^ TAM and UPP1^−^ TAM interactions illustrated the central role of UPP1^+^ TAM in orchestrating cellular communications within the TME (Figure 6M).

## 4. Discussion

In this study, we integrated single‐cell transcriptomics, bulk RNA‐seq, and spatial transcriptomics to systematically characterize TAM heterogeneity in GC. Our analysis identified distinct macrophage subpopulations with tumor‐specific expansion patterns and constructed a robust prognostic signature through machine learning approaches. Furthermore, spatial transcriptomic analysis revealed the metabolic heterogeneity and cellular interaction networks within the TME. These findings provide novel insights into the molecular programs governing TAM function and establish potential therapeutic targets for GC intervention.

The TME in GC is characterized by extensive cellular heterogeneity and complex intercellular communication networks [26–28]. Our single‐cell analysis revealed nine major cell types with distinct transcriptional profiles, consistent with previous reports on GC cellular architecture. Notably, we identified four macrophage clusters significantly expanded in tumor tissues, designated as GC‐associated macrophages. These findings align with emerging evidence that TAMs in solid tumors exhibit substantial heterogeneity beyond the traditional M1/M2 dichotomy. The distinct distribution patterns between tumor and normal tissues suggest that specific macrophage subpopulations are selectively recruited or educated within the malignant microenvironment, potentially driven by tumor‐derived cytokines and metabolic cues.

Through hdWGCNA analysis, we identified nine coexpression modules with distinct expression patterns across macrophage subpopulations. The red, blue, and green modules, specifically enriched in GC‐expanded macrophage clusters, contained genes associated with immune regulation, metabolic reprogramming, and extracellular matrix remodeling. Machine learning‐based prognostic modeling further refined these candidates to a seven‐gene signature (UPP1, VCAN, ELL2, ABCA1, TUBA1A, MX2, and TSPO) with robust predictive performance across multiple independent cohorts. The superior performance of the CoxBoost algorithm, which achieved the highest average AUC values, highlights the utility of gradient boosting methods in handling high‐dimensional genomic data with complex interaction patterns.

Among the prognostic genes identified, UPP1 emerged as the most significant contributor to the risk model. UPP1 encodes uridine phosphorylase 1, a key enzyme in pyrimidine salvage metabolism that has been implicated in tumor progression and chemoresistance in various malignancies [29–32]. Our analysis revealed that UPP1 is predominantly expressed in a specific TAM subpopulation with enhanced interactions with malignant cells, endothelial cells, and fibroblasts. This suggests that UPP1^+^ TAMs may constitute a functionally distinct subset that promotes tumor progression through multiple mechanisms, including metabolic support of cancer cells, angiogenesis induction, and immunosuppression. The metabolic enzyme function of UPP1 raises the intriguing possibility that these macrophages undergo specific metabolic adaptations to fuel tumor growth, warranting further investigation into their metabolic dependencies.

Our data reveal an apparent paradox: UPP1^+^ TAMs correlate with poor prognosis yet also with increased recruitment of CD8^+^ T, Th1, and NK cells. This is not contradictory. First, effector T cells in the TME are often functionally exhausted. Second, UPP1 is a metabolic enzyme that consumes uridine, depriving T cells of essential nutrients and suppressing their function despite their presence [29, 30]. Third, TAMs are highly plastic and may simultaneously produce proinflammatory and immunosuppressive signals. Thus, UPP1^+^ TAMs create a metabolically hostile niche where immune cells are recruited but rendered ineffective.

The spatial transcriptomic analysis provided critical insights into the tissue architecture and metabolic zonation of GC. We observed distinct metabolic profiles across spatial clusters, with specific regions showing enrichment in galactose metabolism, glycerophospholipid metabolism, and amino acid metabolism. This metabolic heterogeneity likely reflects the varying oxygen and nutrient availability across the tumor landscape, as well as the metabolic preferences of different cell populations. The colocalization of UPP1^+^ TAM with malignant cells and the identification of SPON2‐ITGB1 as a top‐ranked ligand‐receptor pair suggest active metabolic and signaling crosstalk within specific tumor niches. SPON2 (spondin‐2) is an extracellular matrix protein that promotes tumor cell migration and invasion through integrin‐mediated signaling, and its interaction with ITGB1 on TAMs may represent a novel axis for therapeutic intervention [33, 34].

UPP1 dysregulation is not restricted to GC. Elevated UPP1 has been reported in glioblastoma (correlating with immunosuppression) [35], breast cancer (promoting premetastatic niche formation via uracil‐fibronectin axis) [30], and pancreatic cancer (supporting uridine catabolism under nutrient stress) [29]. Regarding targeted therapy, no UPP1 inhibitor is clinically approved and no registered trials are ongoing; however, preclinical evidence with the inhibitor 5‐benzylacyclouridine (5‐BAU) in melanoma models suggests UPP1 is a tractable target [36]. These findings support further exploration of UPP1 as a therapeutic and biomarker candidate.

Several limitations of this study should be acknowledged. First, the scRNA‐seq dataset was limited to five paired samples, which may not capture the full spectrum of interpatient heterogeneity. Second, the prognostic model was derived and validated using retrospective public cohorts (TCGA‐STAD and GEO datasets). The retrospective nature of these cohorts may introduce selection bias, and prospective clinical validation is needed to establish the model′s utility for patient stratification. Third, platform heterogeneity and batch effects between different sequencing technologies (e.g., RNA‐seq vs. microarray, different GEO datasets) could affect the robustness of the prognostic signature, although we applied batch correction and independent validation across multiple cohorts to mitigate this issue. Fourth, the spatial transcriptomic analysis was derived from a single dataset (GSE251950) with limited sample breadth and regional coverage; therefore, the spatial interaction networks and metabolic zonation patterns should be validated in larger and more diverse spatial cohorts. Fifth, although our spatial analysis identified candidate interaction networks, functional validation through in vitro and in vivo experiments is necessary to establish causal relationships. Finally, the molecular mechanisms by which UPP1^+^ TAM promote tumor progression, particularly the proposed metabolic competition hypothesis, remain to be elucidated through targeted genetic or pharmacological perturbations.

## 5. Conclusions

In conclusion, our multiomics analysis reveals the heterogeneity of TAMs in GC and identifies a prognostic gene signature with potential clinical utility. The characterization of UPP1^+^ TAM as a functionally distinct subpopulation with context‐dependent immunometabolic activities and dual immunomodulatory functions provides a foundation for developing targeted therapeutic strategies. Future studies should focus on validating these findings in larger clinical cohorts and exploring the therapeutic potential of targeting UPP1^+^ TAM‐mediated metabolic and signaling networks in GC.

## Author Contributions

Zhaoyan Li: conceptualization, review and editing, writing—original draft; Ming Xu: conceptualization and writing—original draft; Yuqing Huang: data curation and visualization; Yuan Wu: methodology and validation; Jiafeng Lu: formal analysis and methodology; Guangtao Zhang: conceptualization, supervision, and visualization; Lan Zheng: funding acquisition, writing—review and editing, and validation. Zhaoyan Li, Ming Xu, and Yuqing Huang contributed equally to this study.

## Funding

This study was supported by National Natural Science Foundation of China (10.13039/501100001809, 82305103); The Shanghai Program for Leading Talents in Traditional Chinese Medicine (Shanghai Municipal Health Commission, [2021] No.2; Traditional Chinese Medicine Scientific Research Project of Jiading District Health Commission, Shanghai (No. 2023‐KY‐ZYY‐02); Traditional Chinese Medicine Scientific Research Project of Jiading District Health Commission, Shanghai (No.2023‐KY‐ZYY‐02); Three‐Year Action Plan for Further Accelerating the Inheritance and Innovative Development of Traditional Chinese Medicine in Shanghai [2025–2027]) (ZY [2025‐2027]‐2‐1‐2).

## Disclosure

All authors reviewed and approved the final version of the manuscript and agree to be accountable for the accuracy and integrity of the work.

## Conflicts of Interest

The authors declare no conflicts of interest.

## Data Availability

The data that support the findings of this study are available from the corresponding authors upon reasonable request.
